# Incorporating terrain specific beaching within a lagrangian transport plastics model for Lake Erie

**DOI:** 10.1186/s43591-021-00019-7

**Published:** 2021-12-11

**Authors:** Juliette Daily, Victor Onink, Cleo E. Jongedijk, Charlotte Laufkötter, Matthew J. Hoffman

**Affiliations:** 1 School of Mathematical Sciences, Rochester Institute of Technology, Rochester, NY United States; 2 Climate and Environmental Physics, Physics Institute, University of Bern, Bern, Switzerland; 3 Oeschger Centre for Climate Change Research, University of Bern, Bern, Switzerland; 4 Institute for Marine and Atmospheric Research, Utrecht University, Utrecht, The Netherlands; 5 Department of Civil and Environmental Engineering, Imperial College London, London, United Kingdom

**Keywords:** The Great Lakes, Beaching, Microplastics, Plastic pollution, Lagrangian transport, Mathematical modeling

## Abstract

Mass estimates of plastic pollution in the Great Lakes based on surface samples differ by orders of magnitude from what is predicted by production and input rates. It has been theorized that a potential location of this missing plastic is on beaches and in nearshore water. We incorporate a terrain dependent beaching model to an existing hydrodynamic model for Lake Erie which includes three dimensional advection, turbulent mixing, density driven sinking, and deposition into the sediment. When examining parameter choices, in all simulations the majority of plastic in the lake is beached, potentially identifying a reservoir holding a large percentage of the lake’s plastic which in previous studies has not been taken into account. The absolute amount of beached plastic is dependent on the parameter choices. We also find beached plastic does not accumulate homogeneously through the lake, with eastern regions of the lake, especially those downstream of population centers, most likely to be impacted. This effort constitutes a step towards identifying sinks of missing plastic in large bodies of water.

## Introduction

Plastic is a ubiquitous source of pollution in various ecological compartments of the world’s oceans and lakes. Historically, researchers have focused on modeling transport of plastic in the open ocean surface and lakes [1, 2]. However, mass estimates of surface plastic based on sampling efforts are orders of magnitude lower than what is predicted by input estimates [3]. Locations of this missing plastic have been proposed, such as suspended deeper in the water column, trapped in the sediment, or that it is filtered out by rivers and does not make it to large bodies of water [4–8]. However, one of the proposed explanations is that this missing plastic remains trapped in coastal zones for extended periods of time, potentially beaching and resuspending before eventually moving to off shore waters [9–12]. Around the world, plastic has been abundantly observed on coastlines, serving as another indicator of the coastline as a proposed reservoir for plastic [13–15]. Coastal zones are also considered to be a major generator of microplastics as the mechanisms present on shorelines are more likely to cause fragmentation [16, 17].

In the Great Lakes, much attention has been devoted to studying the presence of plastic transported in the water and deposited in sediment [7, 8, 18], and these mechanisms have been included in large-scale models for a more complete representation of plastic behavior [2, 19, 20]. Like in the global oceans, plastic has been found on the beaches of the Great Lakes, but specific beaching mechanisms have not been included in any large-scale hydrodynamic models for the lake [13, 18]. Previous modelling efforts in Lake Erie, which include sediment deposition, have shown significant accumulation of particles in the shallow nearshore sediment. This underlines the need to include near-shore processes, such as beaching, in these models for a more accurate understanding of nearshore plastic accumulation [19].

Surface samples taken in the Great Lakes have shown high plastic concentrations, which are even higher than average concentrations in North Atlantic and South Pacific [21–23]. Of the Great Lakes systems, Lake Erie often reports some of the highest surface plastic concentrations [20, 21, 23, 24]. Lake Erie is also an important source of fresh water for the region, and plastic has been found in tap water originating from the lake [25].

The existing work on the beaching of plastic is difficult to compare because of the variety of approaches taken. Beaching research began with a focus on sampling to understand concentrations [13–15]. Hinata et al. [10] expanded on this work to estimated residence times of plastic items on a beach. While the study only considered one beach, it showed various types of plastic items have beach residence times of 69 - 273 days by marking and tracking beached items on the beach over the course of 1 - 2 years.

Preliminary modeling work of beached plastics has not accounted for resuspension [26, 27]. Recently, Onink et al. [11] systematically tested parameterizations for plastic beaching and resuspension on a global scale, identifying coastlines and nearshore water as significant oceanic plastic reservoirs. Currently, there is no modeled plastic beaching work in the Great Lakes. While beaching modeling work specific to plastics is not extensive, we can draw from other fields of particle modeling such as oil beaching [28]. Some observations indicate that different beach types have an impact on beaching and retention of various particles, where areas of more sediment accumulation are more likely to trap particles compared to steep rocky beaches which are less likely to retain plastic [14, 29]. Samaras et al. [30] modeled the behavior of beaching oil droplets and quantified the retention behavior of nine different beach types. However, no similar work has been done to date for microplastic resuspension.

We include our beaching model within a large-scale hydrodynamic model to capture the combined effect of the beaching and open water mechanisms. In this work we incorporate a beaching model from [11] to a previously used hydrodynamic model for Lake Erie [19, 20]. The existing Lake Erie model accounts for three-dimensional advection, diffusion, polymer density and size, and sediment deposition. Additionally, we use a high resolution shoreline classification for the lake to assign terrain specific beaching probabilities. Together this allows us to predict areas of plastic accumulation along the coastline and derive a first pass estimate for the amount of plastic on the beaches of Lake Erie.

## Methods

The hydrodynamic model was previously used in [19], and a two dimensional version was used in [20]. We apply to model to Lake Erie. Lake Erie is the shallowest of the Great Lakes, with an average depth of 19 m [31]. The persistent current in Lake Erie flows west to east with inflow in the west from the Detroit River and outflow in the east to the Niagara River [32]. In the *x*−*y* direction, particle positions are advected given the dynamical system:

$$\frac{dx}{dt} =u(x,y,z,t) $$

$$\frac{dy}{dt} =v(x,y,z,t)$$

where *u*, and *v*, are the interpolated horizontal x-direction, and y-direction velocities, respectively. We assume smooth behavior of currents below grid resolution, which allow for interpolation to the particle location. Here we use cubic interpolation in space, and third-order Lagrange interpolation in time. We solve the system using a Runge-Kutta 4th order numerical scheme (RK4) with timesteps of one hour, and the code is implemented in Matlab.

In the vertical, or *z*, dimension we also model diffusion and density driven sinking in addition to advection. In the *z*-direction, the surface is set to *z*=0 and greater depths have negative values. The vertical position is given by the Milstein solution [33] to an advection diffusion PDE model [34]. This gives *z* as

$${}\begin{aligned} z(t+\delta t)=&z(t)+(w_{a}+w_{b})\delta t+\frac{1}{2}K'(z(t))[\Delta W^{2}+\delta t]\\&+\Delta W \sqrt{2K(z(t))} \end{aligned} $$

where *w*_*b*_ is the rise velocity of the particle, *w*_*a*_ is vertical water velocity, *K*(*z,t*) is the vertical turbulent diffusivity, and *ΔW* is a Gaussian random variable taken from a distribution with mean zero and standard deviation $\sqrt {\delta t}$ for timestep *δt*=5 sec. If a particle moves below the depth of the lake, we consider it deposited and remove it from the system after recording the location.

The rise velocities, *w*_*b*_, were calculated using a modified version of Stokes’ Law to allow for particles of irregular size [35]. With this method, we have a way to calculate sinking velocities for a range of particle sizes, densities, and shapes and also account for changes in sinking velocity due to temperature variations in lake. This method has also been previously used to model microplastics sinking velocities by [36]. Implementing sinking velocity using Stokes’ Equation for particles of irregular shape [35], the velocity is given by

$$w_{b}=\left(\frac{\rho_{p}-\rho_{w}}{\rho_{w}} gw_{*}\nu \right)^{1/3},$$

where *ρ*_*p*_ is the density of the particle, *ρ*_*f*_ is the density of the water, *ν* is the kinematic viscosity of the water, and *w*_∗_, the dimensionless sinking velocity, is given by

$$w_{*}=1.71 \times 10^{-4}D_{*}^{2}$$

with

$$D_{*}=\frac{(\rho_{p}-\rho_{w})}{\rho_{w} \nu^{2}} gD_{n}^{3}.$$

Here *D*_*n*_ is the equivalent spherical diameter, or the diameter of a sphere of the same volume as the particle of irregular shape. To set bounds for *D*_*n*_, we first define the Corey Shape Factor (CSF) as

$$\phi=\frac{c}{\sqrt{ab}}$$

where *a,b*,*c* are the longest, intermediate, and shortest lengths of the particle respectively. We assume *b*=*c*, implying it is symmetric in size along two of its axes. With this assumption,

1 $$  \phi=\sqrt{\frac{c}{a}}  $$

Assuming the irregular particle is an ellipsoid with dimensions *a,b*,*c*, and recalling *D*_*n*_ is the diameter of a sphere with the same volume as the particle of irregular shape:

$$\frac{4}{3}\pi \left(\frac{D_{n}}{2}\right)^{3}=\frac{4}{3}\pi \frac{a}{2}\frac{b}{2}\frac{c}{2},$$

or again assuming *b*=*c* and solving for *D*_*n*_.

$$D_{n}=\sqrt[3]{ac^{2}}$$

Lastly, substituting Equation 1 into the above, we have *D*_*n*_=*aϕ*^4/3^.

An irregular particle presents a worst case scenario for *D*_*n*_, as for a perfectly spherical particle *D*_*n*_ is simply the diameter, so we assume an irregularly shaped particle to calculate a lower bound on *D*_*n*_. To find this lower bound, we use values for CSF from literature, specifically *ϕ*=0.6 which was estimated as the mean CSF for a fragment [37]. Fragments make up 31% of microplastics found in water sampling, the second most common shape after fibers, which represent 48.5% of sampled shapes by count [38]. We do not model fibers because the shape is too irregular to calculate sinking velocity or model as a passive tracer. Additionally, while fibers are common by count, they have a low mass compared to particles are unlikely to account for a significant portion of missing plastic mass [37].

Plastic sample sizes are typically reported as the length of the longest dimension, which here is equivalent to *a*. To generate a range of values for *D*_*n*_, we randomly generate numbers uniformly distributed between *D*_*n*(*min*)_=*a*_*min*_(.6)^4/3^ and *D*_*n*(*max*)_=*a*_*max*_ for whatever range particle size (*a*_*min*_ to *a*_*max*_) we wish to model. Here we model particles with longest dimension from 1.00 mm to 4.75 mm. It is possible that uniform may not be the best distribution for particle size, as sampling efforts tend to find higher quantities of particles at smaller sizes [37]. Investigating different distributions for size could be a potential improvement in future work.

To include beaching, we follow the approach of [11]. We first identify all particles within a 2x2 km grid cell that borders the coastline of the lake as nearshore. The probability of beaching for any nearshore particle is given as

$$P_{b}^{i}=1-\exp{(-dt/T_{b}^{i})},$$

where *dt* is the time step and $T_{b}^{i}$ is the characteristic beaching time at that shore point *i*. Once a particle is beached, the probability of resuspension is given by

$$P_{r}^{i}=1-\exp{(-dt/T_{r}^{i})},$$

where $T_{r}^{i}$ is the characteristic residence time for plastic on the beach for that beach type.

**Fig. 1:**
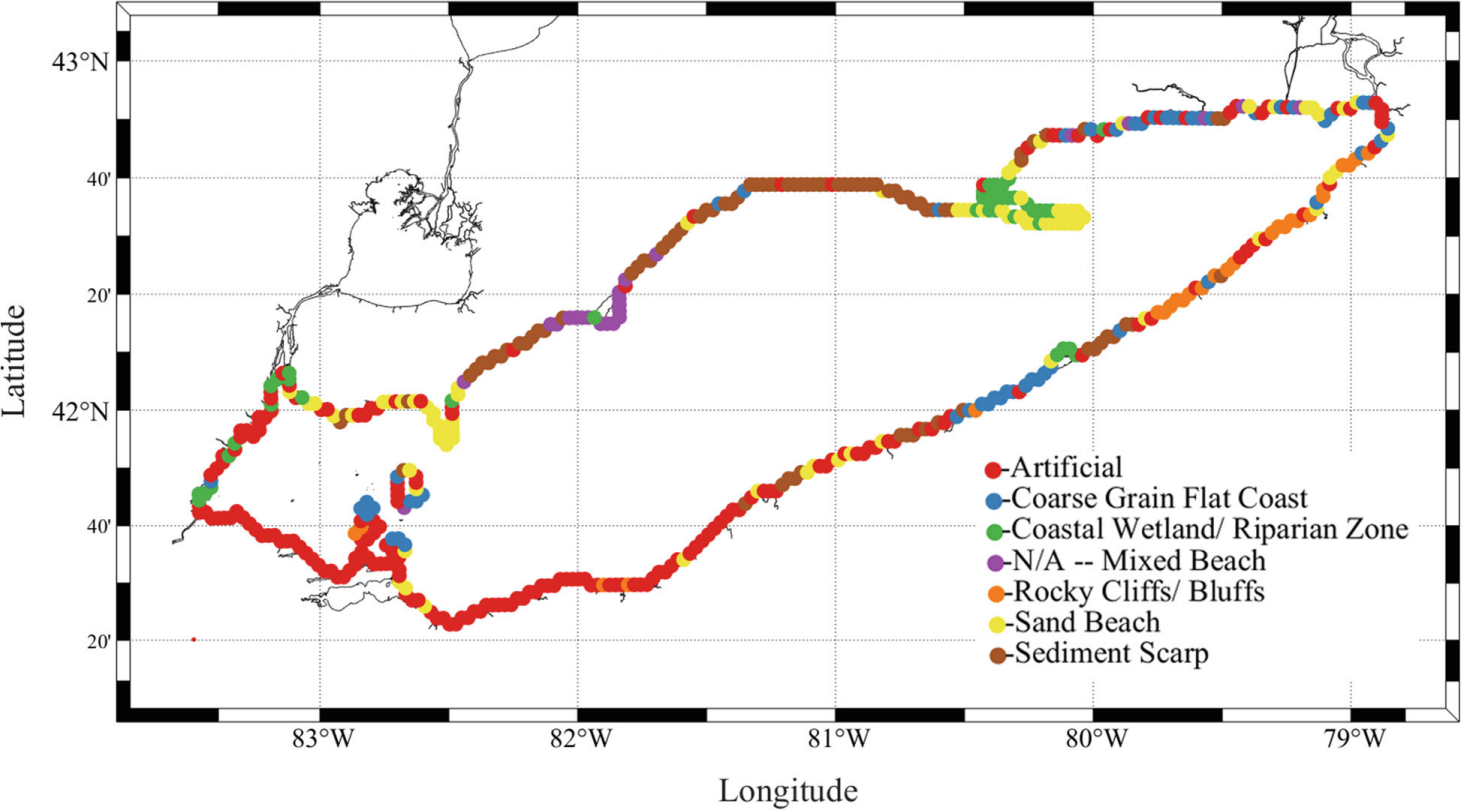
Beach type classifications interpolated to model grid for Lake Erie

We expand the beaching model to include beach type dependence. To classify beach types, we interpolate a beach type data set to our model grid (Fig. 1).We classify seven different beach types of sand beach, artificial, coarse grain flat coast, coastal wetland/riparian zone, N/A – mixed beach, rocky cliffs/bluffs, and sediment scarp (Table 1). These beach types were selected because they were the classification types in the data set, taken from [39]. To include the beach type dependence in the model, we choose $T_{b}^{i}$ and $T_{r}^{i}$ values based on that beach type at shore point *i*. The beaching probability does not depend on changes in the local hydrodynamics, but the stochastic nature of the parametrization is intended to account for this.

**Table 1:** Beaching time and residence time ratios to sand beach for beach type classifications

Erie Classification [39]	Samaras Classification [30]	Beaching time ratio to sand beach $\gamma _{b}^{i}$	Residence time ratio to sand beach $\gamma _{r}^{i}$
Sand beach	Sand beach	1	1
Artificial	Rocky shore	4/3	3/4
Coarse grain flat coast	Sand and gravel	1	1
Coastal wetland/ riparian zone	Sheltered marsh/ mudflat	1/5	5
N/A – Mixed beach	Sand and gravel	1	1
Rocky cliffs/ bluffs	Seawall, concrete, ect.	Inf	0
Sediment scarp	Exposed headland	24	1/24

There is a lack of research on beaching behavior for plastics specifically, so we use ratios, $\gamma ^{i}_{r}$, of the residence time of oil droplets on sand beaches to the residence times for other beach types in the Mediterranean Sea [30]. The beach types in this paper do not directly correspond to the classifications in our data set, so they were paired as accurately as possible, in some cases using satellite images of shorelines to identify characteristics (Table 1). We then use the characteristic residence time, $T_{r}^{sand}$ for plastics on a sand beach from [10] to predict residence times, $T^{i}_{r}=\gamma _{r}^{i}*T_{r}^{sand}$, for all other shore types. We make the assumption for all beach types that the characteristic beaching time is dependent on the reciprocal of the residence time ratio, where $T^{i}_{b}=\gamma _{b}^{i}*T_{b}^{sand}$ with $\gamma ^{i}_{b}=1/\gamma _{r}^{i}$. This assumption is made because if a certain beach type has a high probability of beaching, it is also likely to trap the plastic leading to a long residence time. Conversely, beach types with a low probability of beaching are expected to have a low residence time. This is similar to the approach by [11], where resuspension times were varied using a ratio for the sandiness of a coastline.

To examine the sensitivity of the model we also run a version with no beach type dependence (NBD), meaning a particle has the same probability of beaching or resuspending at any shore point. In the NBD model, the values for *T*_*b*_ and *T*_*r*_ are fixed for the entire lake, so $T_{b}^{i}$ is either 1, 2, or 5 days depending on the run, and $T_{r}^{i}=69$ days. These values are constant for all *i*. This was the lower range of observed residence time for plastics on a beach based on field observations [10], and was also a value used in previous modeling work [11]. The choices for $T_{b}^{i}$ are the lower range of values used in modeling work in the worlds oceans [11]. They were chosen to reflect the lower overall time scales in the smaller system of the lake, as compared to the ocean.

For all models, we prevent nearshore particles from being deposited. This is done to isolate the effect of beaching because as depth goes to zero, we cannot differentiate between beaching and deposition, and deposition is permanent in the model. If model dynamics cause particles to move below the depth in a nearshore cell, they are reset to above the lake floor by a distance of 5% of the lake depth in that spot. We do not anticipate near shore deposition would dramatically impact results, because we model floating polymers that are less likely to sink in the lake.

To input plastic into the model, we release a particle from every nearshore grid point, for a total of 492 particles, and assign each particle a weight representative of the nearshore population at the release point. Nearshore population data comes from [2], and this was also the same method used in [19, 20]. The nearshore population is calculated using US and Canadian census data of postal regions along the lake. We release particles every 12 hours for the first two months of each run. This is done because the modeled distribution of coastal and beached plastic is sensitive to input, and this way we can track the evolution of the distribution without the influence of continuously released plastic. This was also the same approach taken by [11]. These simulations are run with particles of polyethylene (PE) which is positively buoyant, with initial densities, *ρ*_*p*_, randomly sampled from a uniform distribution from 917 to 965 kg/m^3^ [40]. We choose to model polyethylene because it is positively buoyant, meaning unlike a negatively buoyant particle, it will not sink shortly after entering the lake. Floating particles have the opportunity to experience beaching and nearshore dynamics. Polyethylene is also very common; it makes up about 32% of all produced plastic, more than any other single polymer [41].

We use interpolated temperature, diffusivity, and current output from NOAA’s Lake Erie FVCOM hydrodynamic model ran using forcing files from 2012-2014 [42]. FVCOM uses an unstructured grid to fit smoothly to shoreline. For our use, the FVCOM output was linearly interpolated to a regular 2 km spaced grid to reduce computational cost of interpolation within the model. A 2 km grid has been previously used for plastic transport mesh size in Lake Erie [19, 20]. FVCOM is the operational hydrodynamic model used by NOAA Great Lakes Environmental Research Laboratory (GLERL). The density and viscosity of the water, *ρ*_*w*_ and *ν*, are calculated using the state equations for water with salinity zero and temperature output from FVCOM [43].

## Results and discussion

**Fig. 2:**
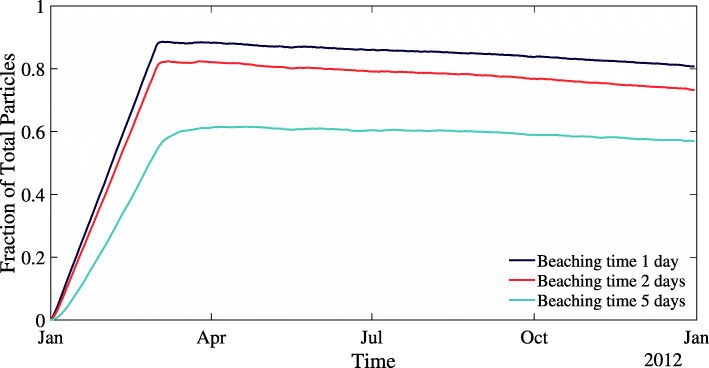
Comparison of influence of three values of *T*_*b*_ on the fraction of beached particles for the model run with no beach type dependence (NBD). Residence time for plastic on the beach was fixed at *T*_*b*_=69 days for all three runs

We first ran model simulations with no beach type dependence (NBD) for a year each, over three runs comparing different parameters. The simulation length was chosen to balance long term model behavior and computational cost while comparing parameter choice. The three choices of 1 day, 2 days, and 5 days for the parameter *T*_*b*_ in the NBD model had a significant effect on the number of beached particles (Fig. 2). With each choice of beaching parameter, the beached fraction increased linearly over the first two months, and then after the particle input ended the beached fraction slowly decreased over time. Lower *T*_*b*_ values reduce the total beached fraction, but the general qualitative behavior remained the same. Because the general behavior is the same for all values of *T*_*b*_, we choose $T_{b}^{sand}=2$ days for the beach type dependence model runs as it is a intermediate choice. The model with beach type dependence is run for three years to capture more long term beaching behavior. We chose three years for the run length because Lake Erie has a hydraulic residence time of 2.7 years [44]. One choice was used for $T_{b}^{sand}$ in the three year run to limit computational cost.

**Fig. 3:**
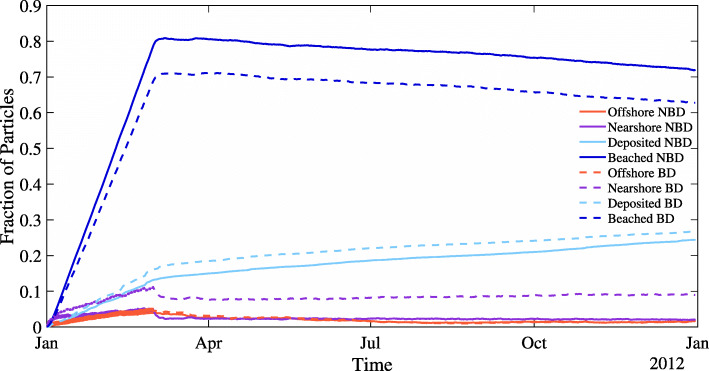
Particle locations over one year for both beach type dependence (BD) and no dependence (NBD) with *T*_*b*_=2 days

We define four reservoirs particles can be in. These reservoirs are beached, deposited, offshore, and nearshore, where nearshore are particles in the adjacent 2 km x 2 km grid cell to shore (Fig. 3). Beached particles make up a majority of all particles after a year long simulation, 62.7% for the model with beach type dependence and 71.9% without beach type dependence. As for differences between the two models, there are slightly fewer beached particles in the beach dependence model because there is, on average, a lower probability of beaching across the lake of.058 versus.061 per three-hour timestep. There is no accumulation of nearshore deposited particles that has been seen in other modeling work because there is no deposition in nearshore grid cells [19]. However, of the reservoirs, the number of deposited particles is the only one with monotonic growth, as it is the only reservoir particles cannot move out of. In this model, deposition is permanent, while in reality particles may have the chance to resuspend. This is a deficiency of the model both as it does not reflect lake dynamics, and if the model were to run indefinitely, all the particles would eventually be in this reservoir.

**Fig. 4:**
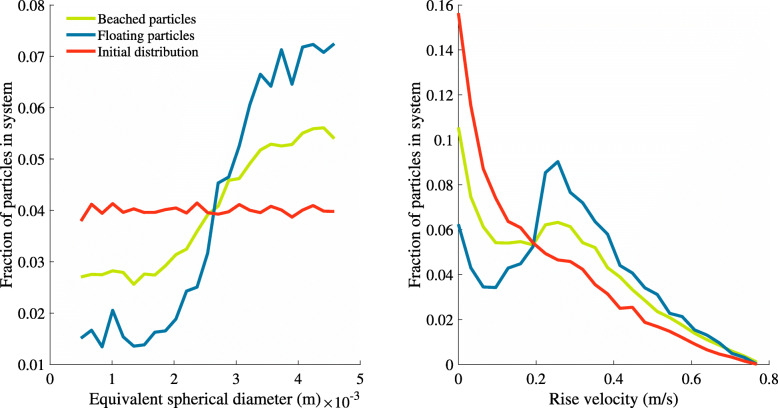
Distributions of particle equivalent spherical diameter, or the diameter of a sphere of the same volume as the particle of irregular shape (left) and calculated rise velocities (right) in the system for beached and floating particles after a three year run. A floating particle has a positive rise velocity

Additional motivation for a more sophisticated sediment resuspension model comes from examining the distribution of particle sizes remaining in the system. Particle density and size begin with uniform distributions. When considering floating, non-beached particles, we see after the three-year run a distinct preference for larger particles to remain in the system (Fig. 4). This is likely due to smaller particles being closer to neutrally buoyant, and thus more likely to move down the water column and ultimately be deposited. This is also consistent with observations by [45], who found fewer smaller microplastics than expected in surface samples, which could be due to increased susceptibility to vertical transport mechanisms. This skew towards larger particles is less noticeable among the beached particles, where the distribution of the diameters is closer to uniform. This is likely because particles can accumulate on the beach where their size and rise velocity becomes meaningless in the scope of our model, and they can not be deposited. Within our model, being beached protects particles from the mechanisms that can introduce a bias towards larger particles. It is possible that if deposition was not an ultimate fate, the size distribution would not be as skewed at the end of the run.

In nearshore water we have removed deposition and replaced it with beaching. However, in the rest of the lake as it is currently implemented, particles are permanently deposited if they hit the lake bed. We also do not account for resuspension from the sediment, which causes the number of deposited particles to increase monotonically. This reduces the number of particles active in the system over time. In a real lake, plastic may resuspend, or move along the lake floor. A more sophisticated model for deposition would ideally incorporate strategies used in this beaching model such as lake bed type specific chances of deposition and resuspension, or consider near-bed velocities and particle transport along the lake bed [46, 47]. However, such data is currently not available and would require additional laboratory and field experiments.

Our model does not account for mechanisms that can remove positively buoyant plastic from the surface, but these mechanisms would also increase depositions. In our model, this would likely have the effect of increasing deposition and reducing the amount of beached plastic. Positively buoyant plastics have been found in samples in both nearshore and deep sea sediment [48]. This is potentially because of biofouling, or the buildup of organic matter and organisms [49]. Biofouling can increase the density of a particle, causing it to sink over time [36, 49, 50]. The role of biofouling could be studied in a future model iteration by combining the current hydrodynamic model with a marine ecosystem model, such as with [51]. The amount of beached plastic could also be influenced by fragmentation, which we do not account for in our model. Fragmentation is the breakdown of the size of plastic particles, often caused by photo-degradation and abrasion [52]. The mechanisms that cause fragmentation can be stronger in shallow, nearshore water, potentially causing fragmentation to have an increased impact on beached plastic [9].

**Fig. 5:**
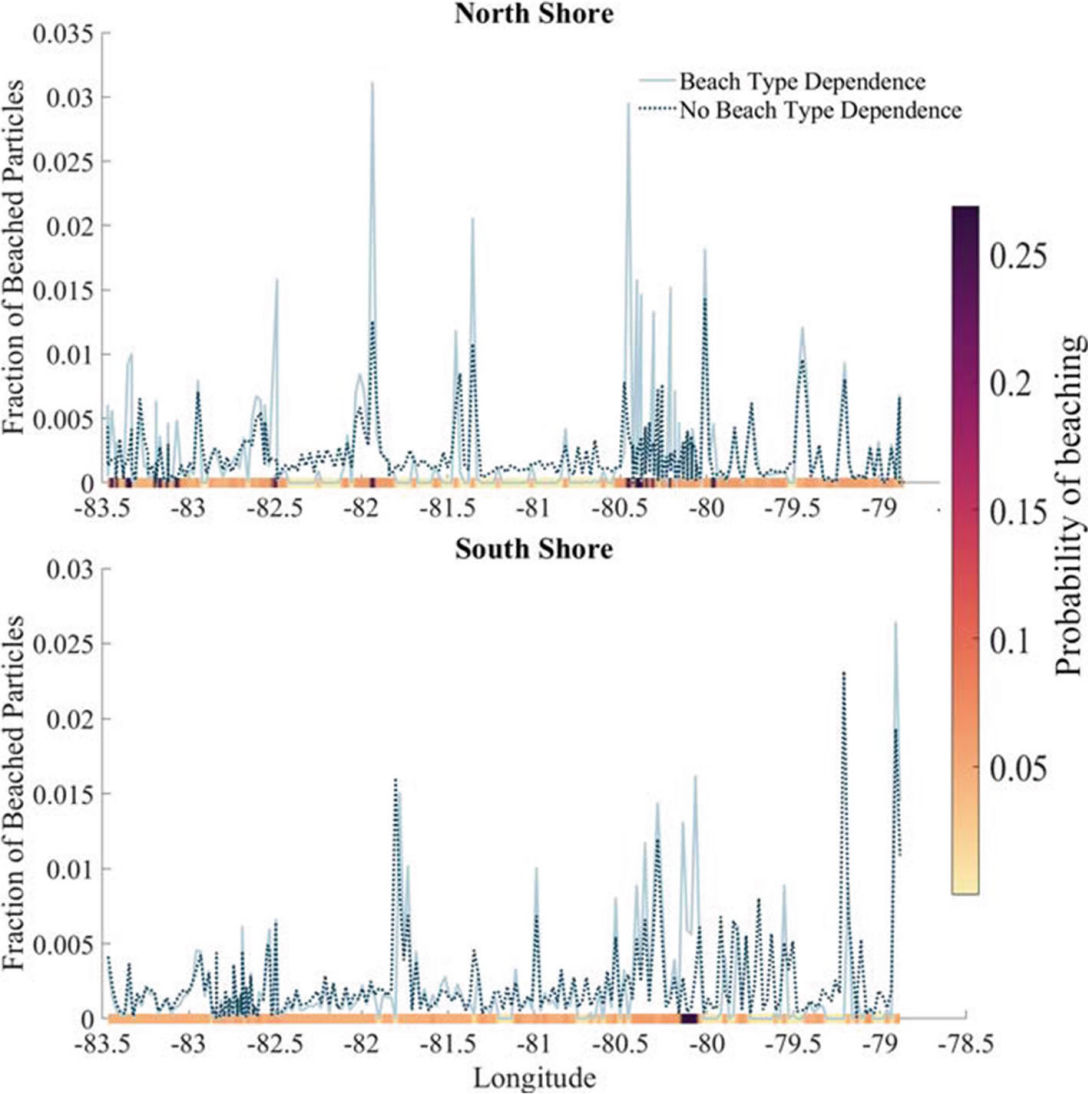
Number of particles beached on the North and South shores for the model with and without beach type dependence. For the model with beach type dependence, probability of beaching is shown on the x-axis. For the model with no beach type dependence, probability of beaching per three hour timestep is.058

Initially when implementing the beach type dependent model, we hypothesized that beach type would be the predominant factor impacting plastic accumulation. It does have an undeniable impact, especially in regions with a high probability of beaching, such as wetlands. However, we still see many similarities between accumulation patterns for both the beach dependence and no dependence models (Fig. 5). These similarities can likely be explained by shore geometry and advection patterns, which are constant through both models. Additionally, some impact from beach type may indirectly be included in the no dependence model. Regions of high sediment accumulation (i.e. sandy beaches or wetlands) have high probabilities of beaching in the model, but also the lake has the physical properties that made this specific beach type in the first place over a much longer timescale, which could also allow for plastic accumulation.

**Fig. 6:**
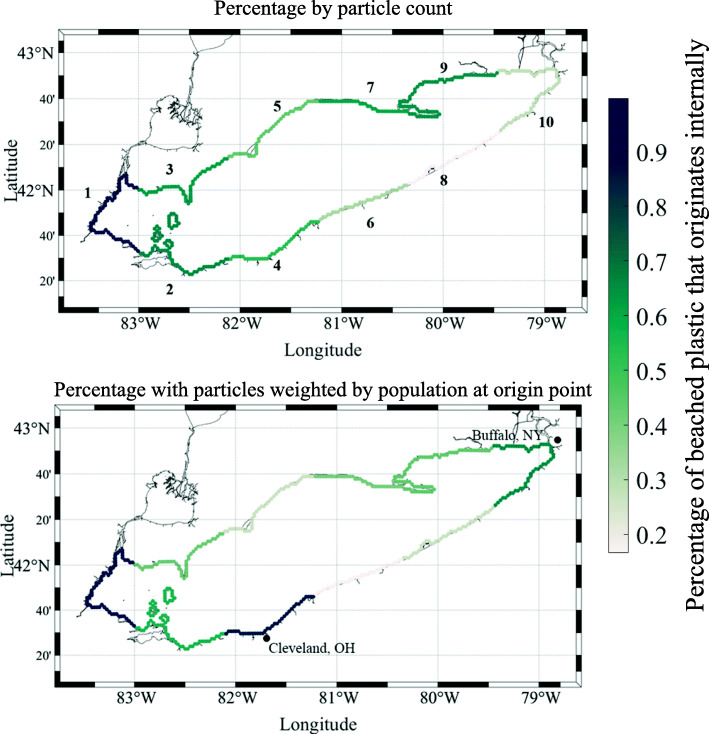
Percentage of beached plastic in a region that originated internally. Top: percentage by particle count, bottom: percentage with particles weighted by population at origin point. Numbers in top figure correspond to region number

The predominantly west to east currents, caused by the prevailing wind and outflow on the east side of the lake, have a large impact on patterns of accumulation and areas with the highest concentrations of beached plastic in the lake. Plastic tends to be pulled towards the eastern side of the lake, which causes plastic in this region to come from all over the lake. Plastic beached at the western side of the lake tends to originate almost entirely from within that region of the lake. When we consider the amount of particles beached by count, this behavior is fairly uniform across the lake, i.e. as we move east, the percentage of beached plastic that originated within that same region drops (Fig. 6). Specifically, in the western most region of the lake (Region 1 in Fig. 6), 100% of beached plastic comes from within that region. Contrasting with this, the eastern most region containing Buffalo, NY (Region 10 in Fig. 6) produced only 41% of the plastic by particle count beached there.

**Fig. 7:**
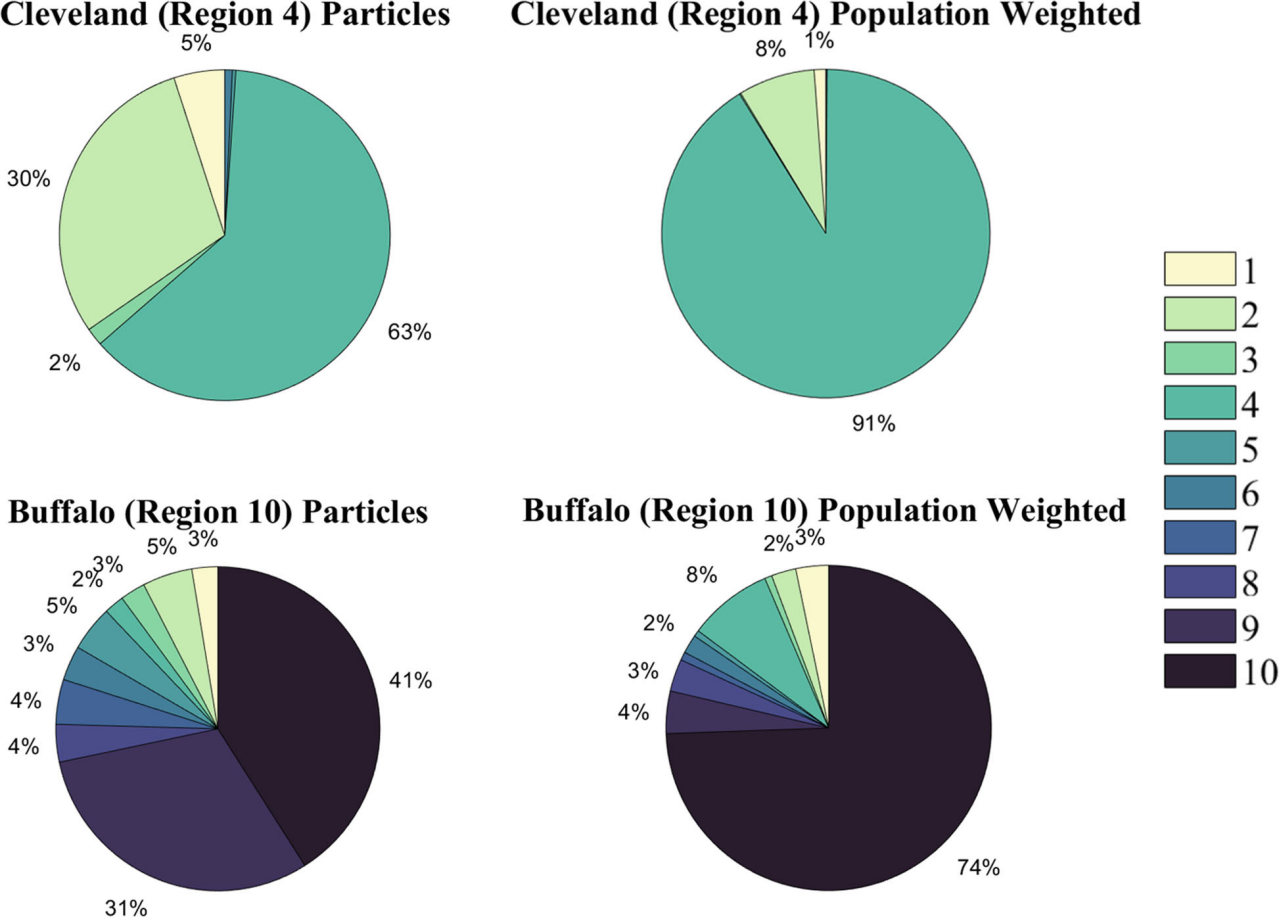
Origins of beached particles in Cleveland (Region 4) and Buffalo (Region 10). Left: percentages by particle count, Right: percentages weighted by population at particle origin point

However, the impact of population centers on the lake can disrupt this trend. If we weight the particles by the nearshore population where they originated, the percentage of plastic in the Buffalo, NY (Population 1.1 million) area that originated internally rises to 74% (Fig. 7) [53]. Additionally, after weighting particles by population, the percentage of plastic in the Buffalo region originating from Cleveland, OH (Population 2.0 million) rises from 2% to 8%. Within the Cleveland region itself (Region 4 in Fig. 6), the percentage of internally produced plastic rises from 63% to 91% after weighting by population. The effect of population centers and prevailing currents can work together to impact regions down current from population centers. In the region immediately to the east of Cleveland (Region 6 in Fig. 6), 54% of the beached plastic originated in the Cleveland region when weighted by population. It is also possible that the beach types across these regions could impact accumulation patterns (Table 2). The coastal wetland beach type only account for 8% of the shoreline in the lake, but holds 29% of beached plastic. These regions likely trap plastic that would otherwise be transported out of that region, and may drive accumulation in the regions they are located such as the western end of the lake (Region 1) or the north east coast (Region 9). Conversely, sediment scarp makes up 17% of the shoreline, but only holds.4% of beached plastic. This may prevent regions with sediment scarp from accumulating self produced plastic, and instead offload it to other regions.

**Fig. 8:**
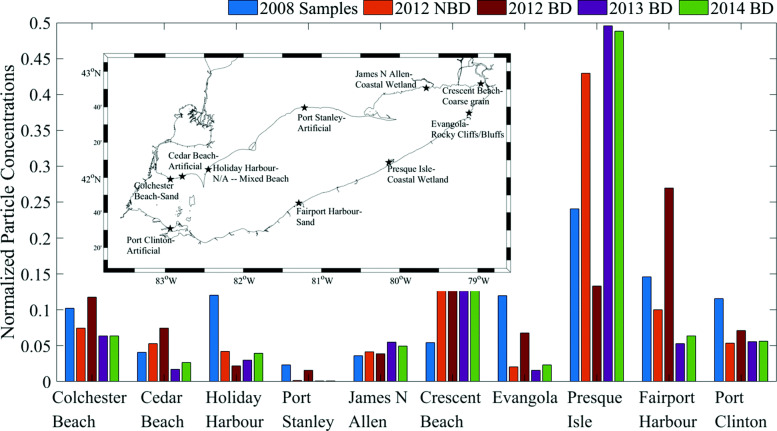
Comparison to 2008 samples [13] for 2012 with no beach type dependence (NBD) and 2012-2014 with beach type dependence (BD). Model classifications of beach type and sample locations shown on the inlaid map

We compare our three year run results, ending in 2013, to the one published beach sample data for Lake Erie that is available in the literature with samples from 2008 [13]. To compare, we normalize our model concentrations and sample concentrations by dividing each by the sum of concentrations of that type (Fig. 8). When considering our model ran with beach type dependence, we see both the highest sample concentration (Presque Isle) and the lowest sample concentration (Port Stanley) agree with the locations of the highest and lowest model concentrations. However, there are some faults with this comparison. The sample locations were all classified as sandy when reported, however the model only classifies shore type down to the grid cell, which is 2 km by 2 km, and does not allow for high enough resolution to capture the full shore complexity. Thus, we see that our model only classifies two of the sample locations as sandy. Additionally, it is especially difficult to compare beached plastic sample data to model results. The beach samples used here, and in general, are normally only taken on sandy beaches. As this is only one beach type, we do not receive data on concentrations for other types to examine model behavior compared to samples in other terrain. Additionally, sand beaches are most likely to be used for recreation, and consequentially more likely to be the site of grooming or trash pickup efforts, which can skew samples collected there. Ideally, the model could be improved by validating with more, taken at regular spacial intervals around the lake to account for all beach types, rather than just sandy. In addition, regularly revisiting beach sites would provide greater insight into the temporal variability of samples concentrations.

**Table 2:** Portion of the shoreline each classification makes up and what portion of beached plastic is beached in that type across the lake

Beach type	Portion of shoreline	Portion of beached plastic
Sand beach	.15	.09
Artificial	.38	.33
Coarse grain flat coast	.11	.21
Coastal wetland/ riparian zone	.08	.29
N/A – Mixed beach	.04	.06
Rocky cliffs/ bluffs	.05	0
Sediment scarp	.17	.004

## Conclusions

In the world’s lakes and oceans plastic mass estimates based off surface sampling differ by multiple orders of magnitude from what is predicted by input estimates, indicating large quantities of missing plastic that are not present at the surface. In the oceans, it has recently been proposed that nearshore beaching plastic is the predominant location of this missing plastic [11, 54–57]. Additionally, previous modeling work for Lake Erie has shown high accumulation of plastic in the sediment in grid cells along the coast, further motivating the inclusion of beaching in the model [19].

Here we model particle beaching within the scope of a three dimensional hydrodynamic model, as the first work for the Great Lakes to do so. Additionally, this is the first large-scale beaching model to include specific plastic beaching probabilities for multiple beach types from broad morphological typologies. The total amount of beached plastic is sensitive to parameter choice for characteristic beaching time, *T*_*b*_, so it is difficult to draw any definitive conclusions about what percentage of plastic litter we expect to be beached in the lake. However, the general accumulation behavior did not show a high dependency on parameters, at least for the parameters tested here. For all the parameter choices we considered, the majority of plastic in the system is beached. We also found that besides shore type, other factors such as advection and shoreline geometry impact accumulation patterns in the lake. We also found that as one moves east across the lake, there is more impact from input from all over the lake, while at the western most side of the lake, 100% of beached plastic is internally produced. We did find that population centers disrupt this general west to east accumulation pattern by causing higher accumulation in their regions, or regions downstream. We would expect comparable results for a similar body of water such as Lake Ontario which has similar size, shape, and prevailing currents as Lake Erie [31]. However, local flow and beach characteristics along with the distribution of population centers can influence beached plastic accumulation.

The parameters used in our model could be improved by additional experimental research on plastic beaching. Additionally, model beaching results are difficult to validate because beach samples often do not reflect the true amount of plastic that is likely to have accumulated. As is the case for Lake Erie, beach samples tend to be taken on sandy beaches [13]. In addition to being unable to compare across beach types, sandy beaches are often used for recreation, and litter is typically routinely removed by grooming or pickup efforts, skewing down the amount of plastic reported in these locations [18]. A sampling effort that took regularly spaced samples around the lake, regardless of beach type, could provide better data for model validation [14].

Beaching plastic results are also heavily dependent on input data, as compared to other plastic modeling, because land-based plastic enters the system directly at the beaching location. In the worlds oceans, land-based plastic is considered the dominant source of plastic pollution [58]. Additionally, while we include population based plastic input from around rivers, we do not specifically model river input as a point source, but rather distribute this input along the coastline near the river mouth. This has the potential to impact accumulation patterns near the river mouth. Wastewater treatment plants (WWTP) are also understood to be a source of microplastics [60, 61]. Additionally, we do not account for plastic released within the lake from fishing or shipping [18]. With a more encompassing input data set, we could likely improve our beaching model and further understand the most impacted areas.

While future work can expand on our findings here, this serves as preliminary model of beached microplastics in Lake Erie. We find that while our parameter choices were uncertain, for the parameters we tested the general behavior of the plastic was similar, with a majority of plastic being beached. The model used here indicates that accumulation in the lake is very dependent on advection patterns, with some impact from shoreline geometry and population centers. In future work we hope to be able to refine parameter choices and include a more complex deposition model.

## Data Availability

The datasets used and/or analysed during the current study are available from the corresponding author on reasonable request.

## Abbreviations

NBD: No beach type dependence
BD: Beach type dependence
